# Supplementary material for: Reconciling Mining with the Conservation of Cave Biodiversity: A Quantitative Baseline to Help Establish Conservation Priorities
Source: PLoS One. 2016 Dec 20;11(12):e0168348. doi: 10.1371/journal.pone.0168348 (PMC5173368; doi:10.1371/journal.pone.0168348)
Supplement: S1 Dataset — (ZIP) [file pone.0168348.s002.zip › Taxa/Serra Norte/SN_2013/N5S/Lista N5S-18.pdf]

CAVIDADE N5S-0018

Classe	Ordem	Fam/Outros	Gên/Outros	Espécie	S-U	U-S	Zon.
Arachnida	Acari	Ixodidae		jovem	X		A
Arachnida	Acari	Opilioacaridae		sp.1	X		E
Arachnida	Acari			jovem	X	X	A
Arachnida	Amblypygi	Phrynidae	*Heterophrynus*	*longicornis*	3	X	
Arachnida	Araneae	Araneidae	*Alpaida*	sp.1		X	
Arachnida	Araneae	Caponiidae		sp.1		X	
Arachnida	Araneae	Corinnidae		jovem	1		E
Arachnida	Araneae	Ochyroceratidae	*Ochyrocera*	sp.1	X	X	P/A
Arachnida	Araneae	Ochyroceratidae	*Ochyrocera*	sp.4	X		A
Arachnida	Araneae	Ochyroceratidae	*Speocera*	sp.1	X		A
Arachnida	Araneae	Oonopidae	aff. *Xyccarph*	sp.2	X		P
Arachnida	Araneae	Theraphosidae		jovem	1		E
Arachnida	Araneae	Theridiosomatidae	*Plato*	sp.1	X		A
Arachnida	Opiliones	Escadabiidae		sp.2	X		A
Arachnida	Opiliones	Neogoveidae		sp.		X	
Arachnida	Pseudoscorpiones	Chthoniidae	*Pseudochthonius*	sp.2	X	X	A
Arachnida	Pseudoscorpiones	Chthoniidae		sp.		X	
Chilopoda	Scolopendromorpha	Cryptopidae	*Cryptops*	sp.		X	
Chilopoda	Scolopendromorpha	Scolopocryptopidae	*Newportia*	sp.		X	
Chilopoda	Scutigeromorpha	Pselliodidae	*Sphendononema*	jovem	3		E/P
Diplopoda	Polydesmida	Fuhrmannodesmidae		jovem		X	
Diplopoda	Polydesmida	Pyrgodesmidae		sp.1	X	X	A
Entognatha	Collembola	Cyphoderidae		sp.1	X		A
Entognatha	Collembola	Paronellidae		sp.4	X		P/A
Entognatha	Collembola	Sminthuroidea		sp.		X	
Entognatha	Diplura	Campodeidae		sp.1	X		E
Insecta	Blattodea			jovem	3		A
Insecta	Coleoptera	Scydmaenidae		sp.4	X	X	A
Insecta	Coleoptera	Staphylinidae	Pselaphinae	sp.12	X		A
Insecta	Diptera	Dolichopodidae		sp.	X		E/A
Insecta	Diptera	Nematocera		sp.		X	
Insecta	Heteroptera	Dipsocoroidea		sp.	X		A
Insecta	Heteroptera	Reduviidae	Emesinae	sp.		X	
Insecta	Homoptera	Cixiidae		sp.	X	X	P/A
Insecta	Hymenoptera	Chalcidoidea		sp.		X	
Insecta	Hymenoptera	Formicidae	*Hypoponera*	sp.2	X		A
Insecta	Hymenoptera	Formicidae	*Pachycondyla*	sp.		X	
Insecta	Hymenoptera	Formicidae	*Paratrechina*	sp.		X	
Insecta	Hymenoptera	Formicidae	*Paratrechina*	sp.1	X		A
Insecta	Hymenoptera	Formicidae	*Paratrechina*	sp.2	X		P
Insecta	Hymenoptera	Formicidae	*Solenopsis*	sp.1	X	X	A
Insecta	Hymenoptera	Formicidae	*Tranopelta*	sp.1		X	
Insecta	Isoptera			sp.	X		P/A
Insecta	Lepidoptera	Noctuoidea		sp.	1		P
Insecta	Orthoptera	Phalangopsidae	*Paraclodes*	sp.	3		E
Insecta	Orthoptera	Phalangopsidae	*Phalangopsis*	sp.	17		A
Gastropoda	Pulmonata	Subulinidae	*Lamellaxis*	sp.		X	
Gastropoda	Pulmonata	Subulinidae		sp.2	X		A
Gastropoda	Pulmonata	Subulinidae		sp.3	X		A

Malacostraca	Isopoda	Scleropactidae	*Circoniscus*	*buckupi*		X	
Symphyla		Scutigerellidae		sp.	X	X	A
Amphibia	Anura	Leptodactylidae	*Eleutherodactylus*	cf. *fenestratus*		X	
Mammalia	Chiroptera	Emballonuridae	*Peropteryx*	sp.		X	
